# Photon-Induced Superior Antibacterial Activity of Palladium-Decorated, Magnetically Separable Fe_3_O_4_/Pd/mpg-C_3_N_4_ Nanocomposites

**DOI:** 10.3390/molecules24213888

**Published:** 2019-10-29

**Authors:** Deepika Thakur, Qui Thanh Hoai Ta, Jin-Seo Noh

**Affiliations:** Department of Nano-Physics, Gachon University, 1342 Seongnamdaero, Sujeong-gu, Seongnam-si, Gyeonggi-do 13120, Korea; thakurdeepz80@gmail.com (D.T.); tathanhhoaiqui2292@gmail.com (Q.T.H.T.)

**Keywords:** nanocomposites, mesoporous g-C_3_N_4_ nanosheets, Fe_3_O_4_ nanoparticles, photocatalysis, antibacterial activity

## Abstract

Three-component nanocomposites (Fe_3_O_4_/Pd/mpg-C_3_N_4_) have been systematically synthesized using a three-step solution method for the photocatalytic bacterial decontamination. The mesoporous g-C_3_N_4_ nanosheets (mpg-C_3_N_4_), which were prepared by the acid treatment, showed a great improvement in photocatalytic performance. The photoluminescence intensity of the mpg-C_3_N_4_ nanosheets was disclosed to drop about 60% from the value of normal g-C_3_N_4_ nanosheets. Decoration of mpg-C_3_N_4_ with palladium (Pd) nanoparticles led to the effective suppression of carrier recombination and the carrier migration to Fe_3_O_4_ nanoparticles. It was revealed that the three-component nanocomposites degraded 99.9% of *E. coli* and 99.8% of *S. aureus* bacterial strains within 2 h of solar light irradiation at a 100 μg/mL concentration, demonstrating their superb photocatalytic antibacterial activity. In addition, the nanocomposites could be easily separated from the bacterial cells and repeatedly used for photocatalytic bacterial degradation with good recyclability. The strong photon-induced antibacterial activity and good recyclability of the three-component nanocomposites may represent their potential as a promising antibacterial photocatalyst.

## 1. Introduction

Environmental protection and wastewater treatment using photocatalysts have been investigated in depth. Particularly, graphitic carbon nitride (g-C_3_N_4_) and its nanocomposites have been adopted for sensing and removal of various toxic pollutants in air and wastewater [1,2]. Despite this advancement, however, there are limited number of photocatalysts that can be applied to inactivate the microbial contaminants by solar light illumination. It is known that the performance of photocatalysts is greatly influenced by their surface area [3], separation of photo-induced charge carriers [4], and exposed reactive sites on the surface [5]. These situations signal the need for a new generation of photocatalysts possessing both highly efficient photoactivity and superior antibacterial activity. To this aim, we have synthesized three-component nanocomposites that can efficiently interact with microbial cells and weaken the cell activity. There have been reported various g-C_3_N_4_-based photocatalysts employing nanoscale heterojunctions with other noble metals such as Au, Ag, Pt, and Pd with differing morphologies [6,7,8,9]. Such noble metals were revealed to increase the lifespan of photogenerated carriers by acting as an electron sink, thus reducing the carrier recombination rate. Among all noble metals, palladium (Pd) is the most attractive, owing to its pronounced synergy effect with g-C_3_N_4_ for enhanced photocatalytic performance [10]. Although those hybrid nanostructures have been commercially utilized, they are not adequate for photokilling of bacterial contaminants, partly due to the limited surface area of the photocatalysts [11]. For this reason, pure g-C_3_N_4_ nanosheets with relatively small surface area and wide band gap may not be an ideal framework for hybridization with noble metals. Morphology-modifying techniques like etching can be utilized to synthesize mesoporous g-C_3_N_4_ nanosheets (mpg-C_3_N_4_), thereby increasing total surface area [12,13]. Furthermore, incorporating Fe_3_O_4_ nanoparticles (Fe_3_O_4_ NPs) as the third structural component, which are magnetically recyclable after use, can allow another positive effects. The Fe_3_O_4_ NPs can play as a supplementary sink for electrons that are migrated from Pd NPs or g-C_3_N_4_ nanosheets. Prior reports on Fe_3_O_4_ NPs-decorated nanocomposites such as g-C_3_N_4_/Fe_3_O_4_/TiO_2_ nanocomposites [14], Fe_3_O_4_/g-C_3_N_4_ nanocomposites [15], and magnetically recyclable ZnO/Fe_3_O_4_/g-C_3_N_4_ photocatalysts [16] demonstrated their photocatalytic performance for removal of toxic dyes and pollutants. However, they have never been explored for removal of microbial contaminants, and their synthesis methods were complex. 

In this work, we synthesized Fe_3_O_4_/Pd/mpg-C_3_N_4_ three-component nanocomposites by a continuous three-step solution method. The nanocomposites were revealed to have strong photoinduced antibacterial activity and magnetic separation capability of microbial contaminants. The as-synthesized nanocomposites showed superior photokilling performance of bacterial strains, proving their potential as a new generation of photocatalysts with low cost, high efficiency, high recovery, and superior antibacterial activity. 

## 2. Results and Discussion

### 2.1. Morphological Characterization of Nanocomposites

**Figure 1:**
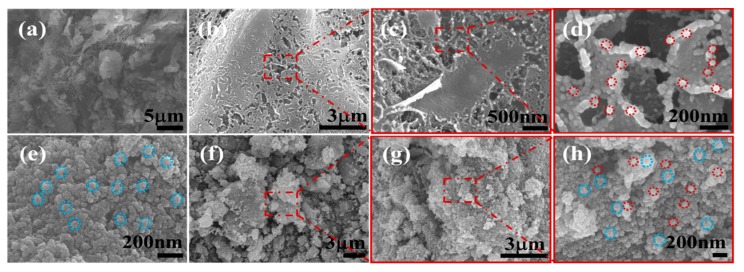
SEM images of (**a**) mpg-C_3_N_4_ nanosheets, (**b**–**d**) Pd/mpg-C_3_N_4_ nanosheets, (**e**) pure Fe_3_O_4_ nanoparticles, and (**f**–**h**) Fe_3_O_4_/Pd/mpg-C_3_N_4_ nanocomposites. Red circles and blue circles represent individual Pd nanoparticles and Fe_3_O_4_ nanoparticles, respectively.

The SEM images of mpg-C_3_N_4_, Fe_3_O_4_ NPs, Pd NPs/mpg-C_3_N_4_ hybrid, Fe_3_O_4_ NPs/mpg-C_3_N_4_ hybrid, and Fe_3_O_4_ NPs/Pd NPs/mpg-C_3_N_4_ nanocomposite are shown in Figure 1. Here, the Fe_3_O_4_ NPs/Pd NPs/mpg-C_3_N_4_ nanocomposite was synthesized by a sequential three-step solution method (see Section 3.2 for more details). At the first step, normal g-C_3_N_4_ nanosheets were transformed to mpg-C_3_N_4_ nanosheets by chemical oxidation with K_2_CrO_7_ solution in H_2_SO_4_. At the second step, Pd NPs were decorated over the mpg-C_3_N_4_ nanosheets using a simple polyol method. Finally, magnetic Fe_3_O_4_ NPs covered the Pd NPs/mpg-C_3_N_4_ hybrid powder by a co-precipitation method. In Figure 1a, the mpg-C_3_N_4_ nanosheets obtained from the acid etching look like flakes with high porosity. These porous g-C_3_N_4_ nanosheets are known to have increased hydrophilicity and large surface area [12]. The increase in hydrophilicity becomes possible by adding hydrophilic functional groups such as –COOH, –OH, and –C=O to the open ends of bulk g-C_3_N_4_ during etching. It was reported that the enhanced specific surface area of mesoporous g-C_3_N_4_ could improve its photocatalytic activity [17]. However, the wide band gap and fast carrier recombination limits the possible use of mpg-C_3_N_4_ nanosheets as novel photocatalysts. Hence, Pd NPs were introduced as a secondary component to retard the carrier recombination. It is found from Figure 1b that the mpg-C_3_N_4_ nanosheets are well decorated with Pd NPs to form Pd NPs/mpg-C_3_N_4_ hybrid nanostructure. From the magnified images in Figure 1c,d, it is confirmed that the small and monosized Pd NPs are coated like a porous film keeping the high porosity of mpg-C_3_N_4_. Although the photocatalytic performance of Pd NPs/mpg-C_3_N_4_ hybrid nanostructure may be improved, its recovery characteristics are poor, limiting its practical applications. Thus, we incorporated magnetic nanoparticles (Fe_3_O_4_ NPs) as the third component. To compare with Pd NPs, the morphology of pure Fe_3_O_4_ NPs is provided in Figure 1e. This image reveals that Fe_3_O_4_ NPs are more rugged and more agglomerated than Pd NPs. The Fe_3_O_4_ NPs are nicely decorated on mpg-C_3_N_4_, as can be seen in Figure 1f. Finally, Figure 1g,h shows that both Pd NPs and Fe_3_O_4_ NPs are evenly distributed over the mpg-C_3_N_4_ nanosheets in a Fe_3_O_4_/Pd/mpg-C_3_N_4_ nanocomposite sample, maintaining the high porosity. To the best of our knowledge, the material design and realization of the Fe_3_O_4_/Pd/mpg-C_3_N_4_ nanocomposite is the first demonstration. 

**Figure 2:**
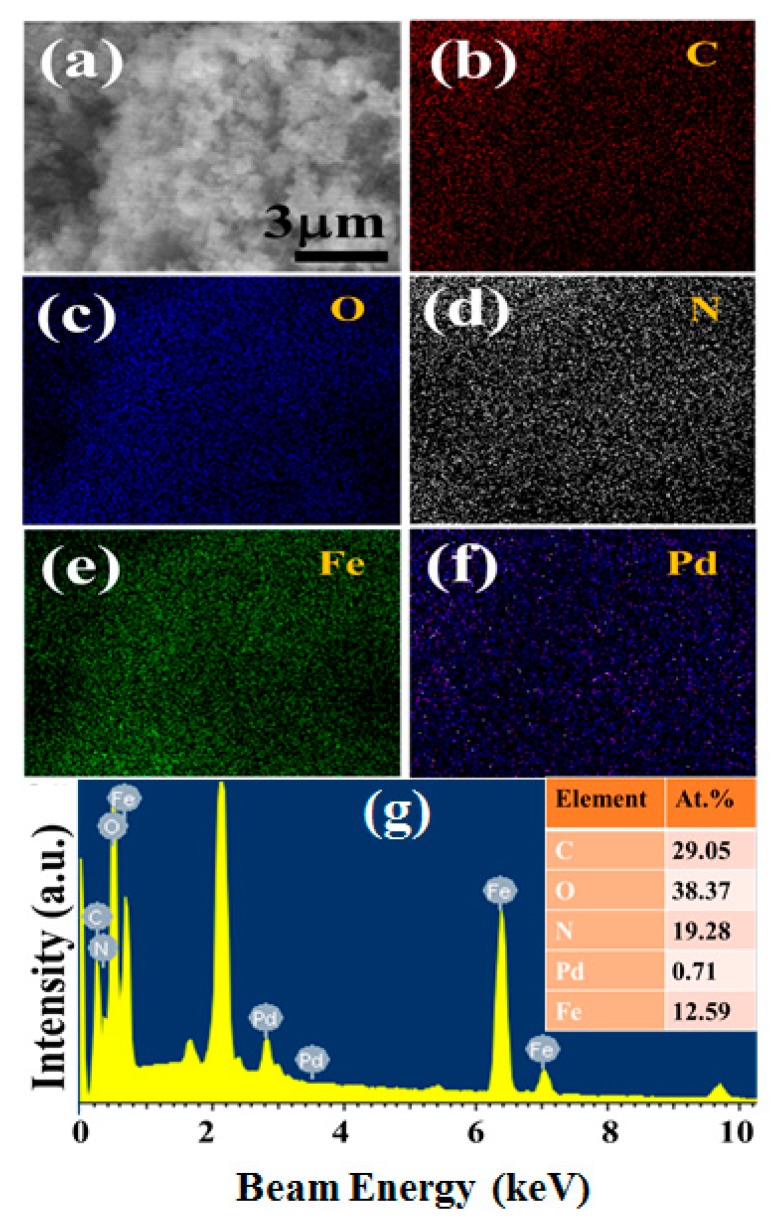
(**a**) SEM image of a Fe_3_O_4_/Pd/mpg-C_3_N_4_ nanocomposite sample. (**b**–**f**) EDX element maps of the sample. The maps are displayed in the order of C, O, N, Fe, and Pd. (**g**) EDX spectrum of the sample.

To further assess the elemental distribution and composition of the nanocomposite, SEM-EDX analysis was performed and its result is shown in Figure 2. From the comparison of real SEM image (Figure 2a) with EDX element maps (Figure 2b–f), it is noted that all the elements are uniformly distributed throughout the nanocomposite sample and no elements other than constituents are observed. This result manifests the high compositional purity and homogeniety of Fe_3_O_4_/Pd/mpg-C_3_N_4_ three-component nanocomposites. Interestingly, the content of Pd NPs is estimated to be only 0.71% for this sample (see the SEM-EDX spectrum in Figure 2g). 

### 2.2. Carrier Separation and Bacterial Degradation 

**Figure 3:**
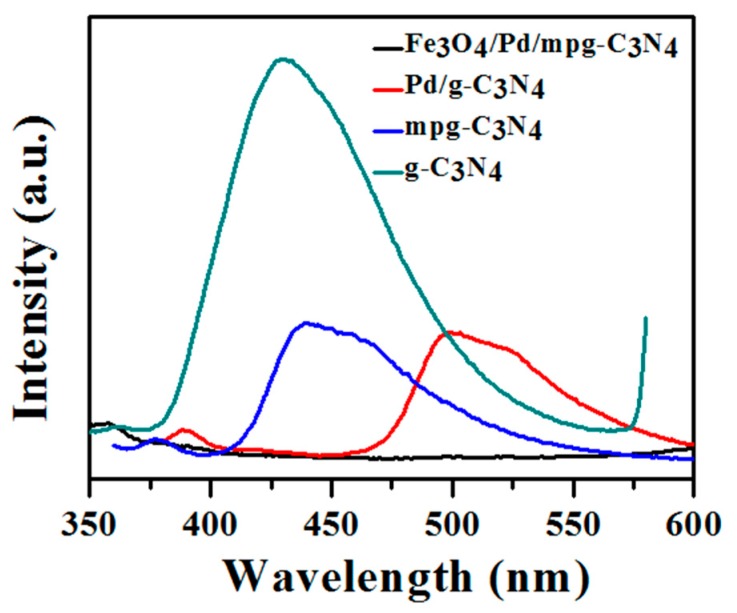
Photoluminescence (PL) spectra of normal g-C_3_N_4_, mpg-C_3_N_4_, Pd/mpg-C_3_N_4_ hybrid, and Fe_3_O_4_/Pd/mpg-C_3_N_4_ nanocomposite.

Suppression of carrier recombination is critically required to enhance the photocatalytic activity of a photocatalyst. As a technique to estimate the carrier separation and recombination rate, photoluminescence (PL) measurements were conducted with excitation wavelength at 320 nm. Figure 3 exhibits PL spectra of normal g-C_3_N_4_, mpg-C_3_N_4_, Pd/mpg-C_3_N_4_ hybrid, and Fe_3_O_4_/Pd/mpg-C_3_N_4_ nanocomposite. The emission peaks of the samples are positioned in the wavelength range of 350–600 nm. The normal g-C_3_N_4_ shows a very strong emission peak centered at 430 nm. The intense PL peak indicates that photogenerated charge carriers massively recombine to disappear in this material. From the peak position, the band gap of normal g-C_3_N_4_ is calculated at 2.88 eV, which is close to its ideal value (2.7 eV). This moderately large band gap is known to lower its light-absorbing capability [18]. It is noteworthy that the PL intensity of mpg-C_3_N_4_ nanosheets is about 60% lower as compared to normal C_3_N_4_, implicating that the high porosity and increased surface area facilitate charge separation. This may be realized because more electrons of photogenerated charge pairs can easily move to larger carrier-depleted surface area. Moreover, a slight red shift of peak position from 430 nm to 440 nm is observed. When the mpg-C_3_N_4_ nanosheets are decorated with Pd NPs, the PL intensity decreases more by ~5% and the red shift becomes noticeable. The additional intensity drop is attributed to electron transfer to Pd NPs and the red shift of the peak position may originate from effective band gap reduction by Pd band formation in mpg-C_3_N_4_ band. Surprisingly, the PL peak intensity seems negligible in Fe_3_O_4_/Pd/mpg-C_3_N_4_ nanocomposite. This substantial intensity reduction suggests that photogenerated electrons in mpg-C_3_N_4_ nanosheets transfer ultimately to Fe_3_O_4_ NPs via Pd NPs and this electron transfer chain works efficiently.

**Figure 4:**
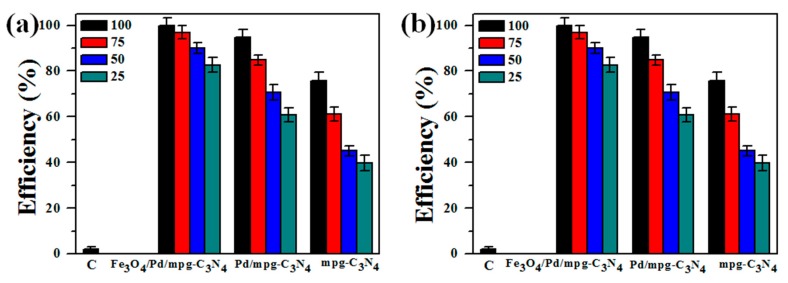
Bacterial degradation efficiency of (**a**) *E. coli* and (**b**) *S. aureus* with 10^8^ CFU/mL. The efficiency of mpg-C_3_N_4_, Pd/mpg-C_3_N_4_ hybrids, and Fe_3_O_4_/Pd/mpg-C_3_N_4_ nanocomposites was estimated at different concentrations (25, 50, 75, and 100 μg/mL) after 2 h of solar light irradiation and magnetic separation. The symbol C represents control sample.

Photodegradation tests were performed on two bacterial strains (*E. coli* and *S. aureus*) for treatment of bacteria-contaminated water. For the tests, the bacteria were diluted to 1 × 10^8^ colony forming unit per milliliter (CFU/mL), and different concentrations of photocatalysts were added and irradiated by solar light (refer to Section 3.3). Figure 4 shows the photon-induced bacterial degradation efficiency of mpg-C_3_N_4_, Pd/mpg-C_3_N_4_ hybrids, and Fe_3_O_4_/Pd/mpg-C_3_N_4_ nanocomposites at different concentrations (25, 50, 75, and 100 μg/mL), employing the standard plate count method. The data were collected after 2 h of irradiation and a control sample was also tested by keeping the sample at room temperature in the dark environment. The bacterial degradation efficiency of *E. coli* and *S. aureus* was calculated by counting the number of colonies using the formula given below:

Degradation efficiency (%) = (*N*_c_ − *N*_t_)/*N*_c_ × 100 (1)

where *N*_c_ is the colony count of control sample and *N*_t_ is the colony count of test sample with *E. coli* or *S. aureus*. In the dark, the bacterial degradation efficiency was estimated below 10% for both *E. Coli* and *S. aureus*. In Figure 4a, the degradation efficiencies of three photocatalysts for *E. coli* are presented as a function of photocatalyst concentration. It is found that the degradation efficiency monotonically increases with increasing the concentration. However, a clear difference is observed in the efficiencies of different photocatalysts at the respective concentrations. The difference is more pronounced at lower concentrations. For instance, at a concetration of 25 μg/mL, the degradation efficiency was estimated at 84.1%, 62.2%, and 41.3% in the order of Fe_3_O_4_/Pd/mpg-C_3_N_4_ nanocomposites, Pd/mpg-C_3_N_4_ hybrids, and mpg-C_3_N_4_ nanosheets. When the conentration was increased to 50 μg/mL, the degradation efficiency was improved to 93.8%, 74.3%, and 47.3%. The rather large difference in efficiency (~20%) between Fe_3_O_4_/Pd/mpg-C_3_N_4_ nanocomposites and Pd/mpg-C_3_N_4_ hybrids indicates the importance of magnetic separation of bacterial species at relatively low photocatalyst concentrations. Finally, the degradation efficiency of Fe_3_O_4_/Pd/mpg-C_3_N_4_ nanocomposites, Pd/mpg-C_3_N_4_ hybrids, and mpg-C_3_N_4_ nanosheets reached 99.9%, 97%, and 81.7% at a 100 μg/mL concentration. Compared with a previous report, in which the photokilling efficiency of pure Fe_3_O_4_ NPs for *E. coli* at the same concentration of 100 μg/mL was 65.6% after 2 h of solar light illumination [19], this degradation efficiency of the nanocomposites represents remarkable progress. Similar trend can be found for *S. aureus*, as shown in Figure 4b. The Fe_3_O_4_/Pd/mpg-C_3_N_4_ nanocomposites, Pd/mpg-C_3_N_4_ hybrids, and mpg-C_3_N_4_ nanosheets exhibited the bacterial degradation efficiency of 99.8%, 94.9%, and 75.8% at a 100 μg/mL concentration. The *S. aureus* cell membrane is thick and its composition also varies depending on the cell wall thickness. For this reason, the comparative efficiency for *S. aureus* is lower than for *E. coli* at every concentration. At lower concentration of 25 μg/mL of catalysts, the degradation efficiency was estimated at 82.7%, 60.9%, and 39.8%, which is slightly lower than the efficiency for *E. coli*. However, the relatively large efficiency difference (~20%) between Fe_3_O_4_/Pd/mpg-C_3_N_4_ nanocomposites and Pd/mpg-C_3_N_4_ hybrids is also found at lower concentrations for this case. 

### 2.3. Photocatalytic Cell Destruction Analysis

**Figure 5:**
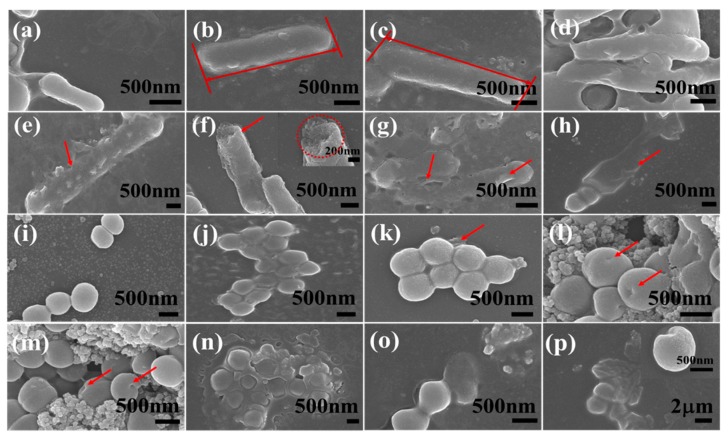
Time-dependent SEM images of *E. coli* (**a**–**h**) and *S. aureus* (**i**–**p**) treated with Fe_3_O_4_/Pd/mpg-C_3_N_4_ nanocomposites (100 μg/mL) under solar light illumination. (**a**,**i**) 0 min, (**b**,**j**) 15 min, (**c**,**k**) 30 min, (**d**,**l**) 45 min, (**e**,**m**) 60 min, (**f**,**n**) 75 min, (**g**,**o**) 90 min, (**h**,**p**) 120 min. The red arrows and a circle point damaged parts of bacterial cells.

The morphological disintegration mechanism of bacteria induced by photocatalysis of Fe_3_O_4_/Pd/mpg-C_3_N_4_ nanocomposites was also studied by SEM. The concentration of the nanocomposites was fixed at 100 μg/mL and solar light was illuminated over a time span of 2 h. The illumination time-dependent cell images are shown in Figure 5a–h for *E. coli* and in Figure 5i–p for *S. aureus*. It is observed that the two bacteria undergo different disintegration mechanisms. The bacterial cell wall is made of lipopolysaccharides, peptidoglycan multilayer, and phospholipid layer, and the wall of *E. coli* cell is thinner (15–20 nm) than *S. aureus* (20–80 nm) cell [20,21]. *E. coli* cell has smooth membrane and rod-shaped morphology before irradiation, as shown in Figure 5a. After 15 min of illumination, the cell shows slight deformation due to the formation and interaction of active species including ·O_2_^−^, *h*^+^, and H_2_O_2_ (Figure 5b). In 30 min, the cell deformation progresses due to the attachment of nanocomposites and attack of more reactive species generated by photocatalysis (Figure 5c). After 45 min of exposure, the *E. coli* cell is mostly covered with nanocomposites and surface interaction goes further (Figure 5d). In 60 min, the cell membrane appears highly damaged, which is responsible for cell death due to the leakage of intracellular materials (Figure 5e). After 75 min of illumination, the ruptured cell membrane provides entrance to the reactive species, which leads to more damage (Figure 5f). From the inset of Figure 5f, a clear hole in the cell wall is found, through which cell materials leak out. The cell is decomposed into its components in 90 min (Figure 5g) and entirely consumed in 120 min (Figure 5h). There has been a report that Fe_3_O_4_/TiO_2_ hybrid nanosheets showed antibacterial activity by solar light irradiation [21]. Considering its result that 4 h of exposure was necessary to completely distort *E. coli*, our complete disintegration time less than 2 h is noticeable.

*S. aureus* bacteria show no photon-induced serious damage in morphology, as shown in Figure 5i–p. The *S. aureus* cell shape is round and its diameter is smaller than *E. coli.* At the beginning (Figure 5i), the cells appear clustered with smooth surface, until the nanocomposites start to interact with the cell surface in 30 min (Figure 5j,k). In Figure 5l,m, curved areas that are pointed by red arrows are found, which may result from the attack of reactive species. The cell damage is not apparent because the nanocomposite has difficulty penetrating the thick cell wall of *S. aureus*. However, the cell cluster appears to be fully dumped into the nanocomposites in 60 min (Figure 5n). Some decomposition of cellular components is noticed in further light exposure, which might be caused by nanocomposite-induced stress to the cell wall (Figure 5o,p). 

### 2.4. Mechanism of Photocatalytic Antibacterial Activity

**Figure 6:**
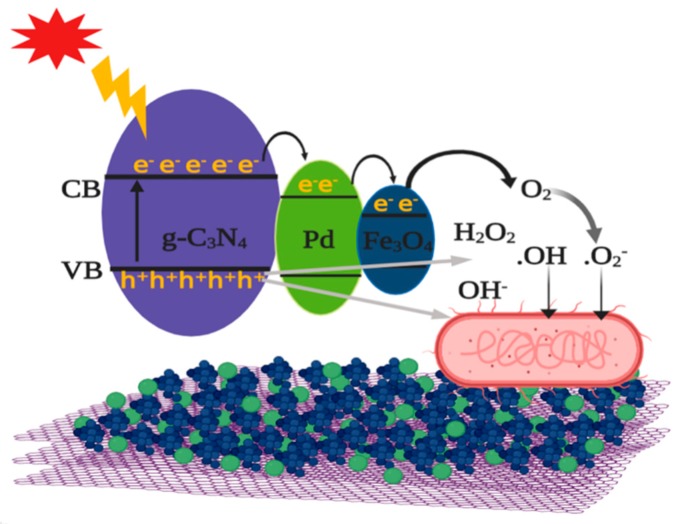
Schematic illustration of photocatalytic antibacterial mechanism of Fe_3_O_4_/Pd/mpg-C_3_N_4_ nanocomposites under solar light illumination.

A schematic illustration of photocatalytic antibacterial mechanism of three-component nanocomposites is presented in Figure 6. When solar light is irradiated, a lot of electron–hole pairs are generated in mpg-C_3_N_4_ nanosheets [22]. Immediately, the photogenerated electrons are transferred to Pd NPs, which facilitate charge separation and also act as stepping stones for further electron transfer. Most of electrons coming into the Pd NPs again move to the conduction band of Fe_3_O_4_ NPs [23]. For this reason, the conduction band of Fe_3_O_4_ NPs would be a main supplier of energetic electrons. On the other hand, photogenerated holes are left behind in the valence band of mpg-C_3_N_4_, due to the energy barrier at interface with Pd NPs. Through these processes, photogenerated charge carriers are well separated, leading to the effective suppression of carrier recombination. Those separated electrons and holes interact with some reactive species such as O_2_, H_2_O_2_, H_2_O, and OH^−^. As a result, superoxide anion radicals (·O_2_^−^) and hydroxyl radicals (·OH), which have hyper-reactivity and strong oxidation capability, are formed and they attack bacteria. Consequently, the oxidative stress at bacterial surface increases and cell materials are damaged, leading to the cell wall disintegration and malfunction, and ultimately to cell death. Through these correlated mechanisms, the three-component nanocomposites can show enhanced antibacterial activity.

### 2.5. Magnetic Separation and Recyclability of Photocatalysts 

**Figure 7:**
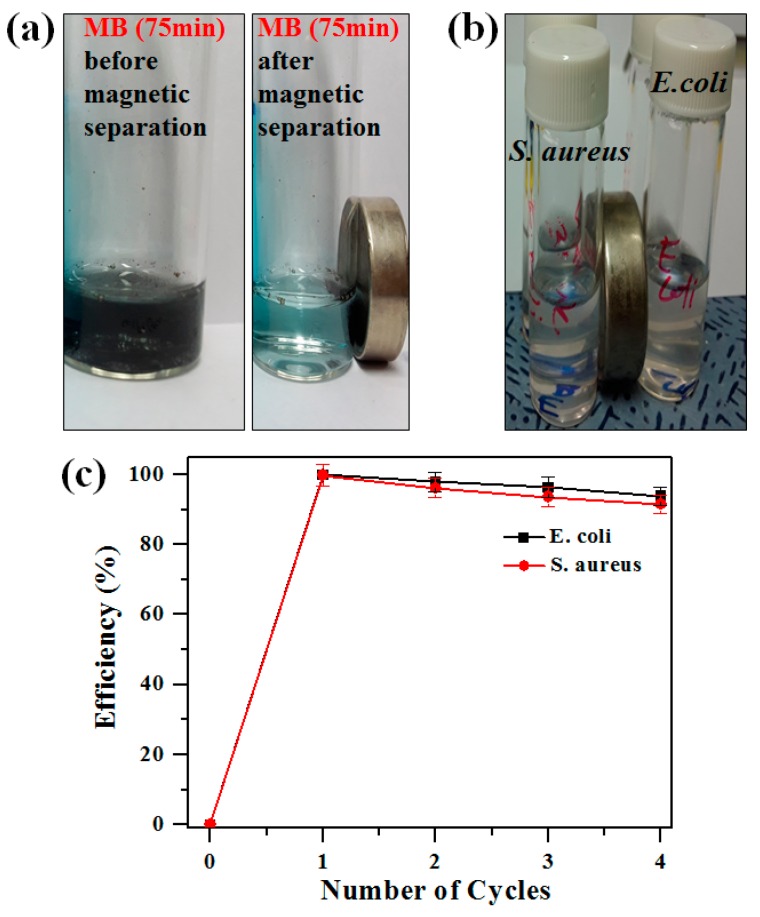
(**a**) Comparison of methylene blue solutions before and after magnetic separation of the nanocomposites after 75 min of light exposure. (**b**) Photo images of bacterial cells (*E. coli* and *S. aureus*) after 10-min-long magnetic separation of the nanocomposites. (**c**) Degradation efficiency of bacterial cells (*E. coli* and *S. aureus*) in the presence of Fe_3_O_4_/Pd/mpg-C_3_N_4_ nanocomposites (100 μg/mL) under solar light irradiation over four cycles of tests. (b) Recovery mechanism of nanocomposite from bacterial cells with magnetic separation for 10 min.

The recovery characteristics and re-usability of photocatalysts were also examined. Due to the strong magnetic property of Fe_3_O_4_ NPs, the Fe_3_O_4_/Pd/mpg-C_3_N_4_ nanocomposites could be easily collected after use, as demonstrated in Figure 7a,b. Figure 7a compares photo images taken before and after magnetic separation of the nanocomposites dispersed in methylene blue solution (10 μM). The images were obtained after irradiating solar light for 75 min. The near-transparent color of the solution confirms that the nanocomposites were completely separated from the solution. The similar and complete magnetic separation of the nanocomposites can also be noticed from bacterial cells, as shown in Figure 7b. To check the recyclability of the nanocomposites, cyclic tests were performed, in which each cycle consisted of photocatalytic bacterial degradation and post-collection of the used nanocomposites. For the tests, the Fe_3_O_4_/Pd/mpg-C_3_N_4_ nanocomposites were repeatedly used while bacterial suspension was refreshed in each cycle. The nanocomposites recovered after each cycle were first sonicated in 75% ethanol solution to get rid of bound bacterial cells, then washed in sterile 0.85% (wt/vol) saline solution for re-use. Figure 7c displays the cyclic degradation efficiency of the nanocomposites for *E. coli* and *S. aureus*. Here, the bacterial cells containing the nanocomposites were illuminated with solar light for 2 h in each cycle. For both bacteria, the change in the degradation efficiency appears to be small. For example, the efficiency drops only by 6% for *E. coli* after four cycles. This small efficiency change along with the quick and easy magnetic separation of the nanocomposites may ensure good recyclability.

## 3. Materials and Methods

### 3.1. Chemicals and Reagents

Iron(III) chloride hexahydrate (FeCl_3_·6H_2_O), iron(II) chloride tetrahydrate (FeCl_4_·4H_2_O), polyvinylpyrrolidone (PVP), melamine (C_6_H_6_N_6_), ethylene glycol (EG, C_2_H_6_O_2_), sodium tetrachloropalladate (Na_2_PdCl_4_), and ammonia (NH_3_) were all supplied from Sigma Aldrich, Ethyl alcohol (C_2_H_5_OH) and acetone were purchased from Daejung Chem. All the chemicals were used as purchased without further treatment. 

### 3.2. Nanocomposite Preparation

**Figure 8:**
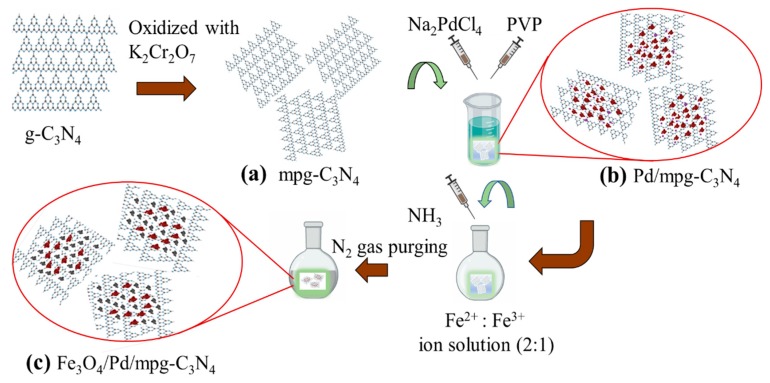
Stepwise synthesis process of Fe_3_O_4_/Pd/mpg-C_3_N_4_ nanocomposites: (**a**) mpg-C_3_N_4_ nanosheets, (**b**) Pd-decorated mpg-C_3_N_4_ nanosheets, (**c**) Fe_3_O_4_/Pd/mpg-C_3_N_4_ nanocomposites.

The nanocomposites were synthesized using an easy, reliable, and continuous three-step solution method. As presented in Figure 8, the first step of nanocomposite preparation was to synthesize mpg-C_3_N_4_ (see Figure 8a). For this, normal g-C_3_N_4_ nanosheets were first prepared by heating melamine at 550 °C for 2 h and ground into fine powder. Then, 500 mg of this powder was chemically oxidized with 8 g of K_2_CrO_7_ solution in H_2_SO_4_ for 2 h at room temperature under continuous stirring. Next, the solution was diluted with deionized (DI) water and residual acids were removed through washing, centrifuging, and drying at 80 °C. At the second step, Pd NPs were decorated to make heterojunctions with mpg-C_3_N_4_ nanosheets by a simple polyol method [24], as shown in Figure 8b. In detail, 10 mL of EG was stabilized at 161 °C for 2 h and then 50 mg of mpg-C_3_N_4_ was added into EG. Subsequently, 0.432 g of Na_2_PdCl_4_ and 0.0832 g of PVP solution in 2 ml of EG was added dropwise over 30 min of time period. The hybrid nanostructure powder was obtained after washing with acetone and drying at 70 °C. At the last step, magnetic Fe_3_O_4_ NPs were decorated on this powder using co-precipitation method in inert gas atmosphere (see Figure 8c), following the previously reported procedure [25]. In order to make Fe_3_O_4_/Pd/mpg-C_3_N_4_, FeCl_2_·4H_2_O and FeCl_3_·6H_2_O (molar ratio = 2:1) were dissolved into 100 mL of DI water and heated at 90 °C for 1 h under nitrogen gas flow. Then, 10 mL of 25% ammonia solution was added slowly. Finally, the black product was magnetically separated, washed, and dried to obtain nanocomposite powder. 

### 3.3. Antibacterial Activity 

The photon-induced antibacterial activity of the three-component nanocomposites at different concentrations (25, 50, 75, and 100 μg/mL) was evaluated by testing two bacterial strains (*E. coli* and *S. aureus*). The bacteria were precultured in LB broth at 37 °C for 15 h and then resuspended in 0.85% saline solution to further dilute to 1 × 10^8^ CFU/mL. Here, varying concentrations of photocatalysts in 0.85% saline solution were tested for 3 mL of bacterial suspension and they were agitated while irradiated by 280 W xenon lamp for 2 h. Simultaneously, another test was also performed in dark circumstance to compare the effect of photocatalysts without light illumination. After this, the photocatalysts were magnetically separated from irradiated suspension simply by placing a magnet nearby for about 10 min. Then, the solution was carefully taken out to investigate bacterial viability after incubating the agar plates at 37 °C for 24 h. The viable colonies were counted by visual inspection, following the standard plate count method.

### 3.4. SEM Observation of Bacterial Morphological Changes

The solar light-irradiated bacterial solution was evaluated for the morphological change caused by photocatalysis with Fe_3_O_4_/Pd/mpg-C_3_N_4_ nanocomposites (100 μg/mL) to better understand photon-induced damage of the bacterial cells. For SEM observation, the bacterial sample was first fixed on the silica glass using 4% of formaldehyde and 2.5% of glutaraldehyde. Then, it was washed by dehydrating the samples into ethanol with the purity of 20%, 40%, 60%, 80%, 95%, and absolute ethanol in a progressive manner for 10 min each. Last, the sample was gold-coated and mounted on the copper grid to observe the morphological changes.

## 4. Conclusions

In this work, Fe_3_O_4_/Pd/mpg-C_3_N_4_ three-component nanocomposites were synthesized by a continous three-step solution method. The mpg-C_3_N_4_ nanosheets were highly porous and both Pd NPs and Fe_3_O_4_ NPs were evenly distributed over the nanosheets. PL measurements confirmed excellent charge carrier separation in the nanocomposites. Notably, the bacterial degradation efficiency of Fe_3_O_4_/Pd/mpg-C_3_N_4_ nanocomposites was predominant over mpg-C_3_N_4_ nanosheets and Pd/mpg-C_3_N_4_ hybrids. In particular, the efficiency reached more than 99% for both *E. coli* and *S. aureus* after 2 h of solar light illumination. Time-dependent photodegradation study of *E. coli* and *S. aureus* demonstrated sequential membrane degradation and cell activity lowering by the attack of highly reactive species. On the basis of roles of respective structural components, the superb photocatalytic and antibacterial machanism of the three-component nanocomposites was proposed. In addition, the nanocomposites showed good recyclability due to the magnetic Fe_3_O_4_ NPs.
